# Uncovering antioxidant and anti-breast cancer potential metabolite from *streptomyces* strain sp. VITAMB using integrated experimental and computational approaches

**DOI:** 10.3389/fphar.2026.1845651

**Published:** 2026-06-23

**Authors:** Arpita Chowdhury, Aparana Kumari, Loganathan Chandramani Priya Dharshini, Abul Kalam Azad Mandal, K. V. Bhaskara Rao

**Affiliations:** School of Bio Sciences and Technology, Vellore Institute of Technology, Vellore, India

**Keywords:** anticancer, antioxidant, breast cancer, diethyl phthalate, paddy fields, *Streptomyces* sp

## Abstract

Actinobacteria’s are rich in bioactive compounds, showing antimicrobial, antiviral, antioxidative, anti-inflammatory, and anticancer activity. The increasing demand for these metabolites in the culinary, pharmaceutical, cosmetic, and nutraceutical industries emphasizes the latent potential of actinomycetes. Paddy field ecosystem provides unique physicochemical conditions that favor the growth of actinomycetes, making them a valuable reservoir of pigment-producing microorganisms. In the present study, a yellow pigment-producing strain identified as *Streptomyces* sp (GenBank ID: OR958670) was isolated from paddy field soil samples collected from Meghalaya. Scanning electron microscopy (SEM) and gas chromatography-mass spectrometry (GC-MS) analyses revealed that this strain produces various metabolites, including diethyl phthalate, which was highlighted following ADME/T (absorption-distribution-metabolism-excretion/toxicity) screening. Diethyl phthalate demonstrated strong antioxidant and cytotoxic activity. In silico studies using a multi-receptor approach assessed the binding energies of diethyl phthalate across several proteins. In silico molecular docking against key regulators of the PI3K/AKT/mTOR signalling axis revealed favourable binding affinities of DEP, with the strongest interactions recorded for PTEN (−7.1 kcal/mol) and AKT1 (−6.9 kcal/mol), both comparable to the positive control Everolimus (−7.2 and −6.6 kcal/mol, respectively), followed by mTOR (−5.2 kcal/mol) and PI3K (−4.8 kcal/mol). Accordingly, the obtained metabolite “diethyl phthalate” demonstrated notable antioxidant activity and moderate cytotoxic effects against MDA-MB-231 breast cancer cells, with an IC50 of ∼153 μg/mL. In addition, docking analysis revealed favourable binding affinities with key proteins associated with breast cancer progression; however, these findings should be regarded as exploratory at this stage, pending validation across a broader panel of cell lines and *in vivo* models. Despite the promising anticancer potential demonstrated in this study, the findings are limited to *in vitro* investigations, and further *in vivo* validation, toxicity assessment, and mechanistic studies are required to confirm the therapeutic applicability of the identified compounds. Future studies should also focus on purification, structural characterization, and clinical evaluation of the bioactive metabolites to facilitate their development as potential anticancer agents.

## Introduction

Actinomycetes are a unique class of filamentous, Gram-positive bacteria that share the traits of bacteria and fungi. These are mostly aerobic, survive in neutral pH environment, and are abundantly found in soil (Shrestha et al., 2021; Pepper and Gentry, 2015). They play an important role in soil biodegradation by decomposing organic debris and aiding in the humus formation. Actinomycetes are known for their geosmin production, imparting the characteristic “wet earth” aroma (Daigham and Mahfouz, 2020). Actinomycetes, under the phylum actinobacteria are a rich source of bioactive compounds with applications in pharmaceutics and agri industry. The genus *Streptomyces* is more significant, contributing to approximately 39% of novel bioactives from actinobacteria (Donald et al., 2022). *Streptomyces* are chemoheterotrophic, that are essential to ecological processes, particularly the turnover of soil nutrients (Hamid et al., 2020). In agricultural environments, like Meghalaya’s paddy fields support a variety of actinomycetes populations, highlighting their significance for preserving soil health and advancing sustainable agriculture (Giri et al., 2020).

Actinomycetes respond to environmental stress by producing a diverse range of bioactive compounds, including those with antibacterial, antifungal, antiviral, antiparasitic, and anticancer properties (Poomthongdee et al., 2015; Basik et al., 2020). These microorganisms possess large genomes, with 5%-10% dedicated to producing secondary metabolites, many of which are used in cancer treatment (Olano et al., 2011). For instance, polyketides like daunomycin and doxorubicin induce DNA cleavage by inhibiting topoisomerase II, while aureolic acids such as mithramycin block RNA synthesis and inhibit topoisomerase I. Nonribosomal peptides, including actinomycin D, interfere with DNA-directed RNA synthesis, and mixed polyketide/non-ribosomal peptides like bleomycin cause oxidative DNA cleavage. Heterocyclic quinones such as mitomycin C cross-link DNA and inhibit DNA synthesis (Olano et al., 2011). *Streptomyces* is a key producer of these anticancer agents, which show strong activity against various cancer cell lines. Granaticins from *Streptomyces sp*. 166 and cervinomycins from *Streptomyces sp*. CPCC 204980 are two examples that showed enhanced cytotoxicity against colon cancer cells. Similar anticancer potential was shown by actinomycin Z6 from *Streptomyces* sp. KIB-H714 and other glycosides, highlighting their significance in clinical study for advanced malignancies (Bahrami et al., 2022).

Cancer remains the foremost causes of death globally even after significant advancements in biomedical research. The most common and deadly of these is breast cancer (BC). Estimates from 2020 GLOBOCAN database stated that female BC accounted ∼11.7% (2.3 million) of all newly reported cancer cases, surpassing lung cancer (Sung et al., 2021). Using data from GLOBOCAN, Kim et al. (2025) conducted analysis that assessed the global burden of female BC in 2022 and forecasted future trends up to 2050 across 185 countries. From Cancer Incidence in Five Continents and WHO database, the study also evaluated 10-year incidence and death trends in 50 and 46 nations, respectively. An estimated 2.3 million new cases and ∼670,000 deaths were reported globally in 2022 alone. While mortality decreased in 29 countries with extremely high HDI levels, and incidence rates rose by 1%–5% per year in almost half of the countries examined. Remarkably, seven countries met the Global Breast Cancer Initiative’s goal of lowering BC by at least 2.5% annually. However, estimates show that the worldwide burden would increase significantly by 2050, with new cases and fatalities predicted to climb by 38% and 68%, respectively, disproportionately impacting low-HDI countries.

Breast cancer arises through a multistep process in which the progressive accumulation of genetic mutations, disrupted hormonal signalling, and immune dysregulation collectively enable tumor cells to proliferate without restraint, evade programmed cell death, and ultimately disseminate to distant sites. The resulting tumor is rarely uniform; instead, it comprises a shifting mosaic of cell populations whose molecular characteristic diverge over time, and this intratumoral heterogeneity is a central reason why conventional therapeutic often loses effectiveness after an initial response. Compounding this challenge, the tumor microenvironment comprising cancer-associated fibroblasts, infiltrating immune effector and suppressor cells, and stromal matrix components, that collectively sustain tumor growth, shield malignant cells from immune recognition, and create conditions that favour disease recurrence. This dynamic microenvironment can blunt the activity of immunotherapeutic agents and serves as a reservoir of signals that drive acquired resistance. The clinical picture is further complicated by the existence of molecularly distinct breast cancer subtypes (e.g., HR+/HER2–, HER2-enriched, triple-negative), each carrying a different prognosis and demanding a different therapeutic strategy. Targeted agents directed against HER2, CDK4/6, PI3K, and PARP have unquestionably improved survival, particularly for patients with metastatic or relapsed disease, yet durable remissions remain elusive for a substantial proportion of patients. A growing body of evidence points to cancer stem-like cells as a key determinant of this therapeutic failure; these cells exhibit intrinsic resistance to cytotoxic and targeted agents, sustain tumor initiating capacity after treatment, and are widely implicated in late recurrence (Xiong et al., 2025). Taken together, these biological and clinical realities underscore the urgent need for alternative therapeutic strategies that can address heterogeneity, circumvent resistance mechanisms, and reduce the toxicity burden associated with prolonged treatment. Natural products have long served as a productive source of clinically useful anticancer compounds. Among naturally derived sources, actinomycetes have proven especially productive, yielding bioactive secondary metabolites of remarakable structural diversity that encompass a wide range of biological activities, and in many cases, more favourable toxicity profiles than synthetic counterparts. Sustained investigation of these natural resources may therefore yield novel therapeutic candidates capable of supplementing or potentiating existing treatment modalities in breast cancer (Longley et al., 2003).

The PI3K/AKT1/mTOR signalling axis is one of the most frequently dysregulated pathways in breast cancer, making it a rational framework for therapeutic targeting (Hao et al., 2025). PI3Ks are membrane-associated lipid kinases activated downstream of receptor tyrosine kinases (RTKs) and G protein-coupled receptors (GPCRs), which catalyze the conversion of PIP2 to PIP3, recruiting AKT to the plasma membrane (Fruman et al., 2017; Vanhaesebroeck et al., 2021). AKT is fully activated via dual phosphorylation by PDK1 and mTORC2, subsequently activating mTORC1 to amplify pro-survival and pro-proliferative signals (Saxton and Sabatini, 2017). PTEN negatively regulates this cascade by dephosphorylating PIP3 to PIP2; its loss in approximately 20%–30% of breast cancers drive constitutive pathway activation and poor outcomes (Millis et al., 2016). Given the high prevalence of PIK3CA mutations (∼30–40% of breast tumors) and clinical approval of alpelisib, capivasertib, and everolimus targeting this pathway, PI3K, AKT1, mTOR, and PTEN were selected as molecular docking targets in the present study.

The current study is centred on the isolation and characterization of actinomycetes from paddy field soil samples in Meghalaya. The investigation identified a purified actinomycetes isolate exhibiting significant antioxidant activity, which was subsequently selected for further examination. This study aims to investigate the antioxidant properties of metabolites derived from *Streptomyces* sp. Strain VITAMB through standard *in vitro* assays, and to evaluate their potential anti-breast cancer activity using *in silico* molecular docking against key breast cancer-associated targets and cytotoxicity assay.

## Methodology

### Sample collection

The soil sample was acquired from a post-harvest wetland paddy field (Latitude: 25° 56′6.162″N; Longitude: 91° 46′14.5524″E) near Umling-Patharkhmah Road, Ri Bhoi District, Meghalaya, India in December. The samples were procured 2–3 inches below the soil surface and transferred to a sterile plastic zip-lock bag. The sample was then taken to the laboratory and was stored at −20  °C until further use.

### Physiochemical analysis of soil

The soil sample was assessed for its physical and chemical characteristics. The physical properties were evaluated by measuring pH with a standard pH meter and determining the moisture content through oven drying. The chemical analysis involved assessing levels of total organic carbon, nitrogen, iron, sulfur, and sulfate. Nitrogen content was determined using the Kjeldahl method (Sáez-Plaza et al., 2013), total organic carbon was measured with the Walkley-Black method (Bahadori and Tofighi, 2016), iron was analyzed with a spectrophotometer (Stalikas et al., 2003), and sulfur and sulfate were quantified using the turbidity method (Wall et al., 1980).

### Isolation of actinomycetes

For the isolation of actinomycetes, 1 g of the soil sample was suspended in 9 mL of distilled water and was serially diluted up to 10^-5^. Further, 100 µL of each soil suspension was added to starch casein agar (SCA) plates (Composition: Ca_3_ (PO_4_)_2_, 0.02 G/L; FeSO_4_⋅7H_2_O, 0.01 G/L; MgSO_4_⋅7H_2_O, 0.05 G/L; KNO_3_, 2 G/L; K_2_HPO_4_, 2 G/L; NaCl, 2 G/L; soluble starch, 10 G/L; casein, 0.3 G/L; agar, 15 g; pH 7.0 ± 0.2) using a sterile glass spreader. To prevent fungal and bacterial contamination, 100 µg of cycloheximide and nalidixic acid, respectively were added to the medium. The culture plates were incubated at room temperature (28 °C ± 20 °C) for 7-14 days.

### Production of bioactive compounds

For metabolite production, the previously screened pure and potential cultures of Actinobacterial isolates were cultured in SCB. The inoculated cultures were incubated at 29 °C for 7-10 days in a shaking incubator at 100 rpm. The formation of visible clumps confirmed growth, and aggregates along with a noticeable increase in turbidity of the culture broth. Following incubation, the culture broths were centrifuged (8000 rpm, 15 min), and the obtained cell-free supernatants were collected for antimicrobial evaluation. Bioactive metabolites from crude were extracted sequentially using methanol, ethyl acetate, and chloroform, in a ratio of 1:1, according to increasing solvent polarity. The mixtures were transferred into a separating funnel, vigorously agitated, and left to stand overnight. The organic layer was separated and subjected for further antioxidant analysis (Banerjee et al., 2024).

### Antioxidant assays

#### DPPH assay and IC_50_ determination

The radical scavenging activity was evaluated using the 2,2-diphenyl-1-picrylhydrazyl (DPPH) method (Kedare and Singh, 2011; Raj et al., 2017). A 2.2 mL of 0.002%, DPPH solution in methanol was mixed with 2.2 mL of different concentrations of crude extract (5-200 µg/mL) in distilled water. For standard antioxidant- ascorbic acid is used, and only DPPH solution served as control. The reaction mixtures were incubated in the dark conditions for 30 min, after which absorbance was measured at 517 nm. The partially purified metabolite was tested at 5, 30, 65, 150, and 200 µg/mL, while ascorbic acid was tested at 20 µg/mL. Radical scavenging activity was calculated using the formula:

Scavenging activity (%) = [(A - B)/ A] × 100, where A is the absorbance of the control and B is the absorbance of the test extract. Data were expressed as mean ± standard deviation (SD) (n = 3).

#### Phosphomolybdenum assay and IC_50_ determination

Total antioxidant activity was assessed using the phosphomolybdenum method (Bokhari et al., 2013; Sahreen et al., 2010). In brief, 1 ml of phosphomolybdenum reagent, 4 mM ammonium molybdate, 600 mM sulfuric acid (H_2_SO_4_), 28 mM sodium phosphate (Na_3_PO_4_) was mixed with 0.1 mL of crude extract. The reaction mixtures were incubated at 95 °C for 90 min, and cooled to room temperature. Absorbance was recorded at 695 nm using a UV-Vis spectrophotometer. The partially purified compound was analyzed at 6.25, 25,5 0, 70, and 100 µg/mL, while ascorbic acid (18 µg/mL) served as the standard. The total antioxidant activity was calculated using the formula:

Total antioxidant activity (%) = [(A0 - At)/ A0] × 100, where A0 is the absorbance of the control, and At is the absorbance of the test extract. Data were represented as mean ± SD (n = 3).

#### Metal chelating activity and IC_50_ determination

Metal chelating activity was determined using the ferrozine–Fe^2+^ complex formation assay (Bhaskara Rao et al., 2014). The reaction mixture containing partially purified extract, ferrous chloride, and ferrozine in a 2:1:4 ratio was incubated at room temperature in the dark for 10 min. Ethylenediaminetetraacetic acid **(**EDTA) served as the positive control, and distilled water was used as the blank. The partially purified compound was evaluated at 10, 30, 55, 90, and 120 µg/mL, whereas EDTA (5 µg/mL) served as the reference chelating agent. Absorbance was measured at 562 nm using a spectrophotometer. Data were expressed as mean ± SD (n = 3).

#### Reducing power assay and IC_50_ determination

The reducing potential of the metabolites was assessed using the potassium ferricyanide reduction method. Briefly, 1 mL of crude extract was mixed with 2.5 mL phosphate buffer and 2.7 mL of potassium ferricyanide, followed by incubation at 50 °C for 20 min. After incubation, 2.7 mL of TCA (trichloroacetic acid) was added, and centrifuged at 10,000 rpm for 10 min. The upper phase (2.7 mL) was carefully collected and mixed with 2.7 mL of distilled water followed by the addition of 0.5 mL of ferric chloride solution. The absorbance was measured at 700 nm. The partially purified compound was tested at 20, 45, 80, 110, and 130 µg/mL, while ascorbic acid (22 µg/mL) was used as the standard. All experiments were conducted in triplicates (n = 3) and results were expressed as mean ± SD.

#### Morphological characterization of potential actinomycetes isolate

The potent isolate was characterized using a range of morphological methods, including growth on various media from the International *Streptomyces* Project (ISP) to assess colony morphology and cultural characteristics. Microscopic observations of mycelial structures and spore chain arrangements were performed using coverslip technique and lactophenol cotton blue staining, observed under oil immersion (100×). Further characterization was conducted using scanning electron microscopy (SEM) (ZEISS EVO18). Spore morphology was evaluated on Starch Casein Agar medium after 7 and 14 days of incubation. For SEM analysis, samples were washed sequentially with 30%, 50%, and 70% ethanol, dried, and coated with gold-palladium using vacuum evaporation before imaging (Buragohain et al., 2023).

#### Molecular identification of the potential isolate

DNA was extracted using a QIAGEN kit in strict accordance with the manufacturer’s instrcutions. Spectrophotometric quantification revealed a DNA concentration of 115 ng/µL, with 260/280 absorbance ratios ranged between 1.9 and 2.0, confirming that the DNA extracted was of high purity for other applications. Purified DNA (7 µL) was analyzed on a 1.2% agarose gel and subsequently used as template for PCR amplification of the 16S rRNA gene using gene-specific primers (Kumari et al., 2026). The PCR conditions included an initial denaturation at 96 °C for 5 min, followed by repeated cycles consisting of denaturation at 96 °C for 30 s, annealing at 50 °C for 30 s, and extension at 60 °C for 1 min and 30 s (Moheet et al., 2015). Phylogenetic analysis of the resulting 16S rRNA sequence was carried out using MEGA-X v10.1.8, while Nucleotide BLAST was used to identify closely related sequences in the database. Multiple sequence alignment was performed using ClustalW, and a maximum-likelihood phylogenetic tree was constructed with 500 bootstrap replications for validation (Bruno et al., 2000).

#### Characterization of active metabolites by gas chromatography–mass spectrometry

The fermented culture was centrifuged at 8,000 and the active metabolites were isolated from the broth using ethyl acetate in a 1:1 ratio with the crude extract reported previously (Kumari and Rao, 2025a). The organic phase containing the metabolites was concentrated under vacuum to yield a dried crude extract, which was then analyzed by GC–MS to profile metabolites secreted by *Streptomyces* sp. Strain VITAMB. GC–MS analysis was performed on a GC-2030 system coupled to an MSQP2020 mass spectrometer (Shimadzu). The instrument is equipped with an HP-5ms capillary column (30 m × 0.25 mm i. d., 0.25 µm film thickness) and was operated in split mode (100:1) with a helium carrier gas flow of 1 mL min^−1^ in constant flow. A 1 µL aliquot of the sample was injected; the column oven was initially held at 45 °C and then ramped at 3 °C min^−1^–280 °C. Detection was carried out in full-scan mode from 50 to 800 amu. Appropriate data-processing and analytical methods were applied to identify and characterize the detected metabolites, ensuring rigorous interpretation of the GC–MS results (Buragohain et al., 2023; Moon and Ku, 2022).

#### *In silico* ADME/T characterization and confirmation through FTIR analysis

The compounds identified from the GC-MS analysis were extracted from the PubChem database (https://pubchem.ncbi.nlm.nih.gov/) (Kim et al., 2019). They were initially screened for ADME/T properties using tools such as SwissADME (http://www.swissadme.ch/index.php) (Daina et al., 2017), Molsoft (https://molsoft.com/mprop/), and ADMETlab 2.0 (https://admetmesh.scbdd.com/service/evaluation/index) (Xiong et al., 2021). Only compounds that adhered to Lipinski’s rule of five, demonstrated favorable ADME profiles, and were non-toxic, non-carcinogenic, and non-mutagenic, were chosen for further molecular docking studies.

FTIR elucidates the functional groups present within the diethyl phthalate. Sample was analyzed using a Thermo Nicolet iS50 spectrometer equipped with an inbuilt ATR, recorded in transmittance mode across 4000-500 cm^-1^.

#### Molecular docking analysis

The three-dimensional crystal structures of PI3K (3O5Z), AKT1 (3O96), mTOR (4JT5), and PTEN (1D5R) were retrieved from the RCSB Protein Data Bank. Missing residues in AKT1, mTOR, and PTEN structures were reconstructed using MODELLER, selecting the best model based on the most negative zDOPE score (GA341 = 1.0). Proteins were stripped of water molecules and heteroatoms, and polar hydrogens with Kollman charges were added before conversion to PDBQT format. A semi-flexible docking protocol was employed, wherein protein structures were treated as rigid while all rotatable bonds of the ligand were kept flexible, enabling full conformational sampling within the binding pocket. Diethyl Phthalate (PubChem CID: 6781) were retrieved from PubChem in SDF format, energy-minimised, assigned Gasteiger charges, and converted to PDBQT format. Docking was performed using SeamDock (AutoDock Vina scoring function) with the grid centred on the co-crystallized ligand coordinates at an exhaustiveness of 30.

To validate the docking protocol, co-crystallized ligands such as MPD (PI3K), IQO (AKT1), P2X (mTOR), and TLA (PTEN), were re-docked as negative controls, while Everolimus (PubChem CID: 6442177) and Paclitaxel served as positive controls for comparative evaluation. Protein–ligand interactions were visualized using PyMOL (Schreodinger, 2020) and Biovia Discovery Studio (Biovia, 2021), with hydrogen bonds identified at donor–acceptor distances ≤3.5 Å and bond angles ≥120°, and hydrophobic contacts assigned at carbon–carbon distances ≤4.0 Å.

#### *In vitro* cytotoxicity analysis

The cytotoxic activity of DEP was determined using the MTT colorimetric assay using MDA-MB-231 cells. The cell line present in this study was obtained from National Centre for Cell Science (NCCS), Pune, India. Cells were maintained in Dulbecco’s Modified Eagle Medium (DMEM) with 10% fetal bovine serum (FBS) and 1% penicillin/streptomycin, and cultured at 37 °C in a 5% CO2 incubator. For the assay, 10,000 cells/well were seeded into 96-well plate and allowed to adhere to the surface. After cell attachment, the culture medium was replaced with 100 µL of complete medium containing varying concentrations of DEP, followed by incubation for 24 h, with each treatment performed in triplicate. Untreated cells served as the negative control, while cells exposed to DMSO serves as the vehicle control. Following the treatment period, 10 µL of MTT solution (5 mg/mL) was added to each well and the plates were incubated for 4 h to allow the formation of insoluble formazan crystals. The resulting crystals were then dissolved by adding 100 µL of DMSO. Absorbance was recorded at 570 nm using a microplate reader (Bukar et al., 2024). Cell viability (%) was calculated from the below given formula:

$$\text{Cell viability }\left(\%\right)=\left(\text{Abs of sample }‐\text{ Abs of blank}\right)\;/\;\left(\text{Abs of control }‐\text{ Abs of blank}\right)\;*100$$

### Statistical analysis

Three independent biological replicates were conducted for each experiment. Data are presented as mean values ± standard deviation (SD). Statistical analysis of the bioassay results was performed using one-way analysis of variance (ANOVA) to determine significant differences among the mean values at a significance level of p < 0.05. Chemical structures and related properties of the compounds were retrieved using online chemical databases. Mean comparisons and statistical analyses were carried out using GraphPad Prism version 8.0.2.

## Results

### Physicochemical analysis of soil sample

With a pH of 7.3 and moisture content of 11.74%, the soil was observed to be neutral. Additional analysis revealed 0.85% total organic carbon, along with concentrations of 1376 mg/kg iron, 736 mg/kg nitrogen, 25.36 mg/kg sulfur, and 76 mg/kg sulfate (SO_4_^2-^) (Table 1). Numerous environmental parameters, such as air conditions, geographic location, soil moisture, soil quality, and pH levels, affect the composition and diversity of bacterial communities (Najar et al., 2022). Supporting this (Liu et al., 2020), stated parameters like pH, nitrogen, phosphorus, and organic carbon, are essential in microbial diversity shaping. Also, we have found in our previous study, the inflated sulfur and sodium levels accord with an increased abundance of Actinobacteria (Kumari and Rao, 2025b).

**TABLE 1:** Physiochemical characteristics of the analyzed soil sample.

Parameter	Unit	Result
Color	-	Dark brown
pH	-	7.3
Temperature	^o^C	11-25
Moisture	%	11.74
Total organic carbon	%	0.85
Iron as Fe	mg/kg	1376
Nitrogen as N	mg/kg	736
Sulphate as SO_4_	mg/kg	76
Sulphur as S	mg/kg	25.36

### Actinomycetes isolation

Actinomycetes were successfully isolated from the paddy soil sample, yielding a total of 20 distinct colonies. All isolates were cultured on SCA and incubated at 28 °C ± 2 °C for 10–15 days under ambient laboratory conditions. Initial screening was conducted on the basis of characteristic colony morphology, with particular attention to the presence of dry, powdery surface growth. Of the 20 isolates recovered, 10 pigment-producing strains were selected for further evaluation of their antioxidant and anticancer potential.

### Antioxidant assays

#### DPPH assay and IC_50_ determination

**FIGURE 1:**
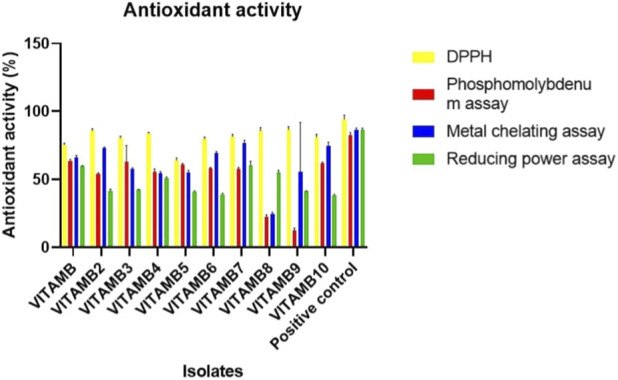
Representation of the different antioxidant activity for the top 10 isolates.

**FIGURE 2:**
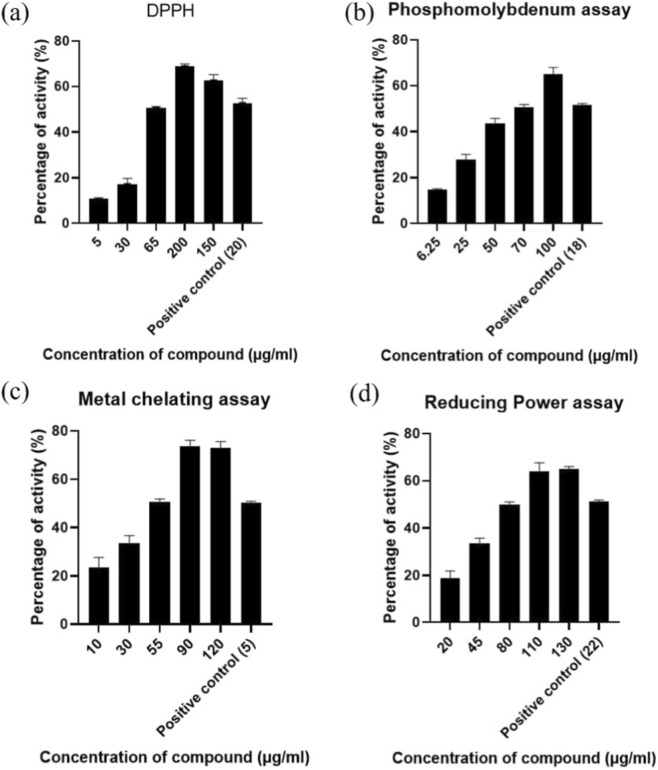
Graphical representation of the IC_50_ values obtained for the compound at different concentrations **(a)** DPPH assay, **(b)** phosphomolybdenum antioxidant assay, **(c)** metal chelating antioxidant assay, and **(d)** reducing power antioxidant assay.

The antioxidant potential of the isolates, demonstrated notable variability in their DPPH radical scavenging capacity. The positive control revealed superior antioxidant activity. Among the tested isolates, VITAMB9 displayed highest radical scavenging activity (86.58%), followed closely by VITAMB2 (85.67%) and VITAMB8 (85.61%). Conversely, VITAMB5 showed the lowest antioxidant activity (64.17%), making it least effective. Other isolates, including VITAMB4 (83.76%), VITAMB7 (81.60%), VITAMB (75.43%), and VITAMB10 (81.12%), demonstrated moderate antioxidant activity. The percentage inhibition observed from DPPH assay is represented in yellow bars in Figure 1. The test compound assessed at varying concentrations (5, 30, 65, 150, and 200 µg/mL), yielded an IC_50_ value of 65 µg/mL. In contrast, ascorbic acid, employed as the positive control, exhibited a significantly lower IC_50_ value of 20 µg/mL (Figure 2a). Consistent with these findings, Janardhan et al. (2014) reported that bioactive metabolites extracted from *Nocardiopsis alba* possess considerable total antioxidant capacity. The analysis revealed antioxidant capacity values of 2.72 ± 0.4, 2.95 ± 1.18, 3.05 ± 0.98, and 1.62 ± 0.4 AA/g for the respective extracts. In comparison, the standard antioxidant, ascorbic acid, demonstrated an activity of 10.63 ± 0.85 AA/g. Research conducted by Das et al. (2024) investigated the antioxidant activity of Myrica esculenta. Antioxidant activity was measured using DPPH, ABTS, and FRAP assays. Additionally, the highest FRAP activity was observed in the methanol extract, measuring 87.125 ± 0.33 mg Trolox equivalents (TE)/g. The results showed that the methanol extract displayed strong antioxidant activity, with IC_50_ values of 22.27 ± 0.98 µg/mL for DPPH and 19.69 ± 0.36 µg/mL for ABTS assays, indicating greater potency compared with ascorbic acid. Furthermore, the FRAP activity was highest in the methanol extract, measuring 87.125 ± 0.33 mg TE/g.

#### Phosphomolybdenum assay and IC_50_ determination

In the phosphomolybdenum assay, the isolate VITAMB demonstrated a moderate level of total antioxidant capacity, (62.6%–64.67%). This performance of VITAMB below the strongest antioxidant-producing isolates in the assay but still within a stable and consistent activity range. Compared with the highest-performing samples, which recorded antioxidant percentages above 80%, VITAMB achieved an average antioxidant capacity of approximately 63.72%, indicating a modest yet reliable contribution to total antioxidant activity. The red bars in Figure 1 depict the percentage activity corresponding to the phosphomolybdenum assay. Ascorbic acid, used as the standard reference antioxidant, exhibited an IC_50_ value of 18 µg/mL, whereas the partially purified compound showed an IC_50_ value of 70 µg/mL, as presented in Figure 2b.

#### Metal chelating assay and IC_50_ determination

The metal chelating assay results indicated substantial differences among the isolates. Positive control displayed highest chelating efficiency (86.44%), which corresponded with its markedly low absorbance. VITAMB9 (77.38%) and VITAMB10 (77.26%) demonstrated highest chelating potential. VITAMB7 also showed strong chelating ability, with an activity of 76.84%. Other isolates such as VITAMB, VITAMB2, and VITAMB6, exhibited moderate chelation capacity, with activity ranging from 66.18% to 72.91%. In contrast, VITAMB8 showed the weakest chelating activity (24.69%). The blue bars in Figure 1 represent the percentage activity associated with the metal chelating assay. For C_50_ determination, EDTA was used as the reference chelating agent and exhibited an IC_50_ value of 18 µg/mL. In comparison, the partially purified compound achieved 50% metal ion chelation at 55 µg/mL (Figure 2c).

#### Reducing power activity and IC_50_ determination

The reducing power assay results demonstrated clear variation in the electron-donating capacity. The positive control showed highest reducing potential (86.39%), while the control group displayed negligible activity. Among the isolates, VITAMB displayed a moderate reducing ability (59.69%), indicating a stable but intermediate capacity for electron donation. The reducing power assay was illustrated as green bars in Figure 1. In the IC_50_ analysis, ascorbic acid showed an IC_50_ value of 22 µg/mL, while the partially purified compound reached an IC_50_ of 80 µg/mL (Figure 2d). The consistent performance of VITAMB suggests that it contributes reliably to the overall antioxidant profile of the isolate group.

### Characterization of isolates

#### Growth in various medium

Based on the results of antioxidant activity, one potential isolate was characterized using various ISP media as described in Table 2. Except for ISP 2, the isolate displayed substantial growth on all other ISP media. The table showed the growth of *Streptomyces* sp. In various media, each with different nutritional compositions. Pronounced growth (+++) was observed in ISP1 (Tryptone Yeast Extract Agar), ISP3 (Oat Meal Agar), ISP4 (Inorganic Salts - Starch Agar), ISP5 (Glycerol Asparagine Agar Base), ISP6 (Peptone Yeast Extract Iron Agar), and ISP7 (Tyrosine Agar). The robust growth observed across multiple media formulations indicates that the isolated *Streptomyces* sp. Possesses considerable metabolic versatility, with a demonstrated capacity to utilize a broad spectrum of carbon and nitrogen sources, including peptides, amino acids, starch, glycerol, and tyrosine. This growth pattern reflects the organism’s adaptability to both nutritionally simple and compositionally complex substrates. Notably, the absence of growth (-) on ISP2 (Yeast Extract - Malt Extract Agar) suggests that the isolate may have a limited capacity to metabolize malt extract, or alternatively, that these medium lacks one or more nutritional components essential for its growth. Taken together, the growth profile observed across the ISP media series underscores the metabolic flexibility of the *Streptomyces* sp. And its ability to thrive under varied nutrient conditions, reflecting the diverse biochemical capabilities characteristic of this genus.

**TABLE 2:** Growth of the potential isolate in various mediums (ISP).

ISP1	++++
ISP2	-
ISP3	++++
ISP4	++++
ISP5	+++
ISP6	++++
ISP7	+++

++++ = Good growth; +++ = Moderate growth; + = Poor growth; - = no growth.

#### Morphological characterization of potential actinomycetes isolate

**FIGURE 3:**
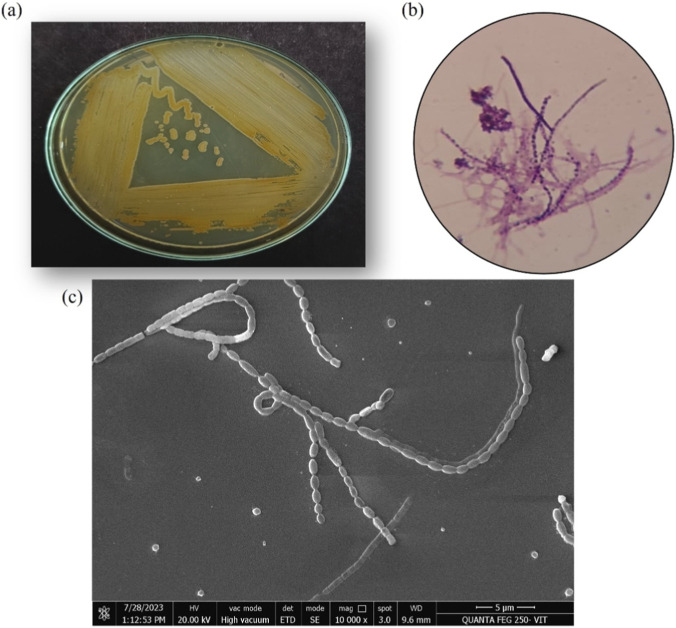
Morphological characterization of the potential isolate- **(a)** Pure isolate on SCA, **(b)** Microscopic view under 100x (Gram Staining), **(c)** SEM view under 13500x magnification.

The potential isolate was morphologically characterized after it was purified and cultured on SCA medium. Gram staining was used to confirm the presence of Gram-positive filamentous structures when observed under 100× magnification. In addition, SEM analysis revealed that the isolate exhibits a recti-flexible spore-chain morphology at 13,500× magnification (Figure 3).

#### Molecular identification and phylogenetic evaluation of the potential isolate

**FIGURE 4:**
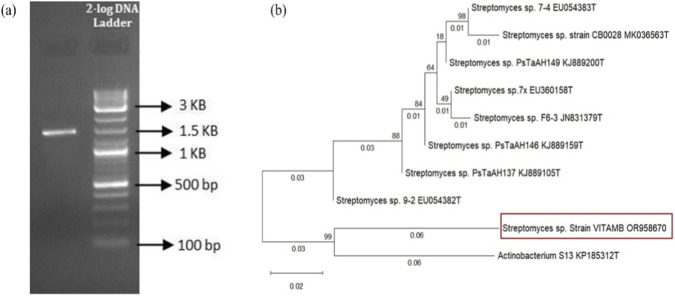
Molecular characterization of the potential isolate- **(a)** Gel electrophoresis illustration, **(b)** Phylogenetic tree of potential isolate.

The extracted genomic DNA and the gel electrophoresis results are shown in Figure 4a. The microbe was identified as *Streptomyces* sp. Strain VITAMB based on its 16S rRNA gene sequence (GenBank ID: OR958670). The sequence shows closest similarity to *Streptomyces* sp. 7x (Sequence ID: EU360158) with 98.42% sequence similarity. Phylogenetic analysis reveals that the strain VITAMB forms a distinct branch within the *Streptomyces* clade, indicating its phylogenetic distinctness. The phylogenetic relationship among these strains is illustrated in Figure 4b, where *Actinobacterium* S13 (KP185312) has been used as the out-group.

#### Metabolite production of potential isolate

Starch casein broth was used as the culture medium, maintained at a pH of 7.0 ± 0.2, to support the bulk production of the microbial isolate through fermentation at room temperature. During fermentation, the cell pellets settled at the bottom of the medium and exhibited a distinct circular morphology. The supernatant obtained from the culture showed an orangish-yellow colour. This supernatant was then processed through solvent extraction to prepare it for Gas Chromatography–Mass Spectrometry analysis.

#### Characterization of active metabolites by gas chromatography–mass spectrometry

**FIGURE 5:**
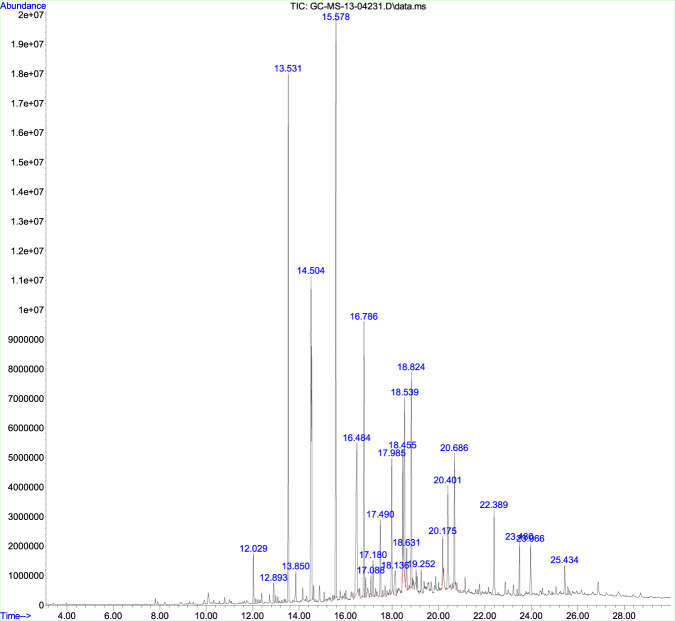
Chromatogram peaks obtained from GC-MS analysis.

Gas Chromatography–Mass Spectrometry analysis was carried out to identify and characterize the chemical constituents of the active metabolite isolated from *Streptomyces* sp. Strain VITAMB. The analysis revealed a total of 25 compounds. The most abundant components detected in the crude pigment were phenol, 2,4-di-tert-butyl-6-nitro- (15.67%), DEP (14.40%), and phenol, 2,4-bis-1,1-dimethyl ethyl (12.5%). The chromatogram peaks (Figure 5) were examined to determine the chemical profile of the pigment, including the types of compounds present and their relative abundance, which are presented in Table 3. In other study, GC–MS analysis of *Streptomyces sparsus* VSM-30 identified several secondary metabolites, including hydrocarbons, phthalate derivatives, peptides, and imidazole compounds. These bioactive constituents indicate that the ethyl acetate extract may exhibit antibacterial, antifungal, and antioxidant activities (Managamuri et al., 2017). Phthalic acid esters (PAEs) have been detected in several natural sources such as plants, algae, and microorganisms, indicating may not solely be a human-made pollutant (Huang et al., 2021). The compounds identified through GC–MS were tentatively assigned based on spectral matching with the NIST library and retention characteristic. FTIR analysis was carried out to determine the major functional groups present in the compound through their characteristic absorption bands. However, detailed structural characterization using NMR spectroscopy was not included in this study.

**TABLE 3:** Comparative chemical composition of metabolite produced by *Streptomyces* sp. Strain VITAMB.

Peak	Retention time	Compound identified	Compound classification	% Composition
1	12.029	1-Tetradecene	Unsaturated aliphatic hydrocarbon	1.15
2	12.893	2-Acetylfluorene	Polycyclic aromatic hydrocarbon	0.64
3	13.531	Phenol, 2,4-bis(1,1-dimethylethyl)-	Phenol	12.15
4	13.85	Pentaerythritol tetraacetate	Polyol	0.88
5	14.504	Diethyl phthalate (DEP)	Phthalate ester	14.4
6	15.578	Phenol, 2,4-di-t-butyl-6-nitro-	Phenol	15.67
7	16.484	Tetradecanoic acid	Saturated fatty acid	7.97
8	16.786	1-Octadecene	Long chain hydrocarbon	7.29
9	17.088	i-Propyl 12-methyl-tridecanoate	Fatty acid methyl ester	0.59
10	17.18	Octacosanol	Fatty alcohol	0.97
11	17.49	Phthalic acid, isobutyl octyl ester	Pthalic acid ester	2.11
12	17.985	2-Methyl-6-tert-octylphenol	Octylphenol	3.79
13	18.136	Dibutyl phthalate	Phthalate ester	1.36
14	18.455	1,2-Benzenedicarboxylic acid, butyl 2-methylpropyl ester	Phthalic acid ester	2.72
15	18.539	n-Hexadecanoic acid	Saturated fatty acid	6.11
16	18.631	Phthalic acid, 2-cyclohexylethyl butyl ester	Pthalic acid ester	1.15
17	18.824	Cycloeicosane	Cyclic hydrocarbon	5.93
18	19.252	Tricosyl trifluoroacetate	Monocarboxylic acid anion	0.65
19	20.175	cis-Vaccenic acid	Long chain fatty acid	1.1
20	20.401	Octadecanoic acid	Saturated fatty acid	3.08
21	20.686	1-Docosene	Unsaturated hydrocarbon	3.57
22	22.389	10-Heneicosene	Cyclic hydrocarbon	2.74
23	23.48	Bis-2-ethylhexyl phthalate	Phthalate ester	1.3
24	23.966	Cyclotetracosane	Cyclic hydrocarbon	1.64
25	25.434	1-Nonadecene	Long chain hydrocarbon	1.01

#### ADME/T analysis of the GC-MS compounds

**FIGURE 6:**
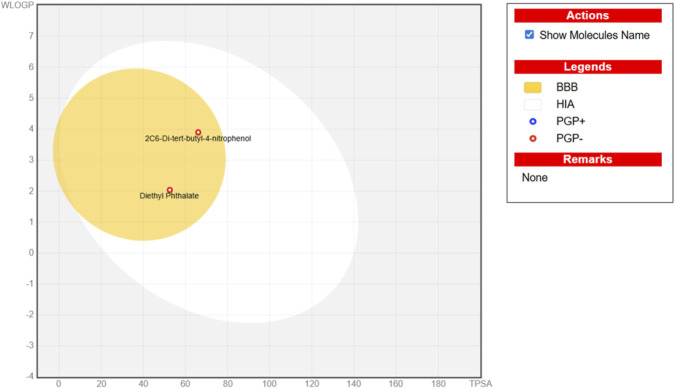
Boiled egg model illustrating the predicted gastrointestinal absorption and BBB permeability of 2,4-di-t-butyl-6-nitro- and diethyl phthalate.

Among the several compounds identified through GC–MS analysis, 2,4-di-t-butyl-6-nitro- (PubChem ID: 69765) and DEP (PubChem ID: 6781) satisfied Lipinski’s rule of five and demonstrated acceptable ADME/T profiles (Tables 4, 5). The compound DEP is characterized by a molecular weight of 222.24 g/mol, a Log P value of 2.39, six rotatable bonds, zero hydrogen bond donors, four hydrogen bond acceptors, and a TPSA of 52.6 Å^2^, exhibiting favourable drug-like properties within the permissible limits. Furthermore, DEP showed a bioavailability score of 0.55 (predicted oral bioavailability ∼14.4%), high gastrointestinal (GI) absorption, good blood–brain barrier (BBB) and water solubility (Figure 6). It was also predicted to be a non-inhibitor of CYP2D6 and displayed higher plasma clearance (8.023) with a markedly low AMES toxicity probability (0.036), indicating minimal mutagenic risk.

**TABLE 4:** Lipinski’s analysis for the target compounds.

Compound	Pubchem ID	Lipinski’s rule	Molecular weight (g/mol) ≤ 500	Lipophilicity (log P ≤ 4.15)	Rotatable bonds ≤ 10	Hydrogen bond donor < 5	Hydrogen bond acceptor < 10	Total polar surface area (< 140 Å^2^)
2,4-Di-t-butyl-6-nitro-	69,765	No violations	251.32	3.17	3	1	3	66.05
Diethyl phthalate	6781	No violations	222.24	2.39	6	0	4	52.6

**TABLE 5:** Tabular representation of target compounds for ADME/T analysis.

Compound	Pubchem ID	Absorption		Distribution		Metabolism	Excretion	Toxicity
		Bioavailability	GI absorption	Water solubility	BBB penetration	CYP2D6 inhibitor	CL_plasma_	AMES toxicity
2,4-Di-t-butyl-6-nitro-	69,765	0.55	High	Soluble	Yes	No	7.458	0.547
Diethyl phthalate	6781	0.55	High	Soluble	Yes	No	8.023	0.036

Although 2,4-di-t-butyl-6-nitro- satisfies Lipinski’s criteria, several unfavourable pharmacokinetic and safety parameters limited its suitability as lead candidate, such as higher lipophilicity (Log P: 3.17), greater TPSA (66.05 Å^2^), lower plasma clearance (7.458), and a higher AMES toxicity probability (0.547). Owing to these less desirable properties, the compound was not selected for further investigation. Collectively, these characteristics indicated that DEP represented a more suitable candidate for further docking analysis.

#### FTIR confirmation of diethyl phthalate

**FIGURE 7:**
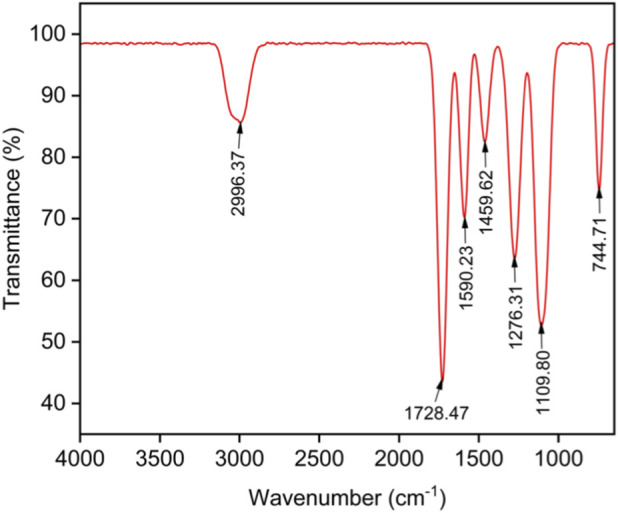
FTIR spectrum of diethyl phthalate (DEP) recorded in the range of 650-4000 cm^−1^.

The FTIR spectrum of DEP confirmed its structure through key characteristic absorptions (Figure 7). The medium band at 2996.37 cm^−1^ corresponds to C–H stretching of the ethyl ester groups (–OCH_2_CH_3_), and the absence of a broad O–H band (∼3200–3550 cm^−1^) confirms complete esterification. The strongest absorption at 1728.47 cm^−1^ is assigned to the aromatic ester C=O stretch, slightly red-shifted from aliphatic esters due to conjugation with the benzene ring. Bands at 1590.23 cm^−1^ and 1459.62 cm^-1^ are attributed to aromatic C=C ring stretching and–CH_2_– scissoring, respectively. The strong absorptions at 1276.31 cm^−1^ and 1109.80 cm^−1^ correspond to C–O–C asymmetric and symmetric stretching of the ester linkage, and the band at 744.71 cm^−1^ confirms the 1,2-disubstituted (ortho) benzene ring through aromatic C–H out-of-plane bending, distinguishing DEP from its isophthalate and terephthalate isomers.

#### Molecular docking analysis

The four target proteins such as PI3K, AKT1, mTOR, and PTEN are central regulators of the PI3K/AKT/mTOR signalling axis, each possessing distinct catalytic domains and binding site topologies. PI3K (PDB: 3O5Z) harbours an ATP-binding cleft within its catalytic subunit, lined by key residues GLU18, TRP56, and TYR74, forming a hydrophobic pocket amenable to competitive binding. AKT1 (PDB: 3O96) is a serine/threonine kinase whose ATP-binding pocket within the kinase domain is delineated by residues TRP80, THR211, and TYR272, mediating hydrophobic and hydrogen bonding interactions. mTOR (PDB: 4JT5) possesses a deep hydrophobic catalytic cavity defined by TYR2225, TRP2239, VAL2240, CYS2243, and MET2345, governing ligand selectivity and accommodation. PTEN (PDB: 1D5R) is a dual-specificity phosphatase with a positively charged active site pocket shaped by the conserved P-loop and critical residues ARG172, TYR176, GLU291, GLU329, and PHE324, coordinating substrate binding and phosphate transfer. These structurally distinct binding sites formed the basis for evaluating the molecular interactions of DEP across the PI3K/AKT/mTOR pathway.

**FIGURE 8:**
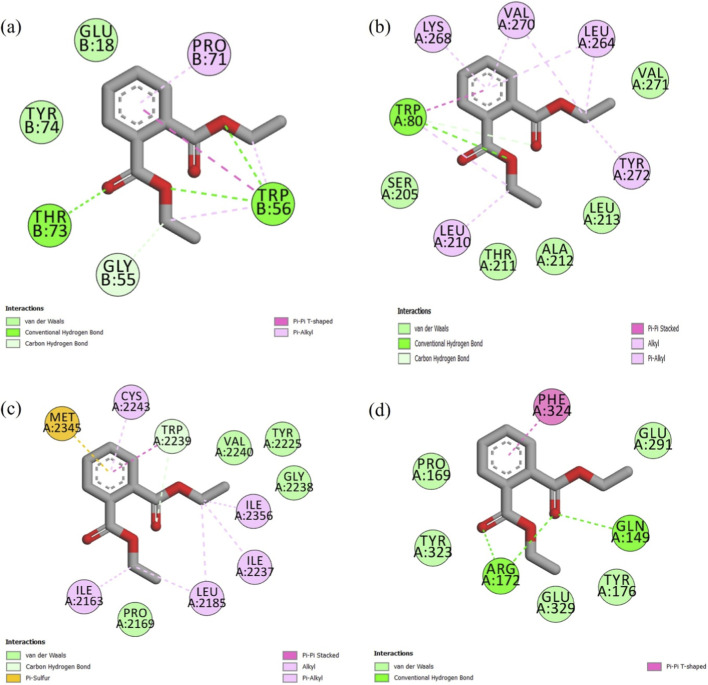
Interactions of DEP (target compound) with BC progression associated proteins- **(a)** PI3K-DEP, **(b)** AKT1-DEP, **(c)** mTOR-DEP, and **(d)** PTEN-DEP.

Molecular docking was performed using a semi-flexible protocol, wherin protein structures were treated as rigid while the rotatable bonds of DEP were kept flexible, enabling full conformational sampling within each binding pocket- MPD (PI3K), IQO (AKT1), P2X (mTOR), and TLA (PTEN), were re-docked under identical parameters as negative controls to validate the docking protocol, while Everolimus and Paclitaxel served as paclitaxel controls. The comparative binding affinities of DEP alongside all controls are presented in Table 6, and the detailed residue-level interaction profile of DEP with each target is summarized in Table 7, with corresponding 2D interaction diagrams illustrated in Figure 8.

**TABLE 6:** Molecular docking binding affinities-comparative analysis.

Protein name	PDB ID	Negative control (kcal/mol)	Everolimus (kcal/mol)	Paclitaxel (kcal/mol)	DEP (kcal/mol)
PI3K	3O5Z	−3.5 (MPD)	−6.4	−8.7	−4.8
AKT1	3O96	−15.1 (IQO)	−6.6	−10.3	−6.9
mTOR	4JT5	−6.8 (P2X)	−7.8	−10.6	−5.2
PTEN	1D5R	−5.5 (TLA)	−7.2	−10.4	−7.1

**TABLE 7:** Protein selection and docking scores between target compound with breast cancer-associated proteins.

Protein name	PDB ID	Mutation & organism	Resolution	Ligand/molecule	Ligand coordinates	Binding affinity (kcal/mol)	Hydrogen bond residues	Hydrophobic and other contacts
PI3K	3O5Z	No, *Homo sapiens*	2.01	Yes	X: 40.928 Y: 5.868 Z: 18.180	−4.8	GLU18, TYR74, THR73	Pi-pi T-shaped: TRP56; pi-Alkyl: PRO71; vdW: GLY55
AKT1	3O96	No, *Homo sapiens*	2.70	Yes	X: 8.351 Y: 6.882 Z: 12.595	−6.9	TRP80, SER205 THR211	Pi-pi stacked: TYR272; pi-Alkyl: LYS268; vdW: LEU210, ALA212, LEU213, VAL270, VAL271, LEU264
mTOR	4JT5	No, *Homo sapiens*	3.45	Yes	X: 51.885 Y: 0.004 Z: 49.163	−5.2	CYS2243 TYR2225	Pi-sulfur: MET2345; Pi-Alkyl/Alkyl: TRP2239, VAL2240, ILE2237, ILE2356, LEU2185, ILE2163, PRO2169; vdW: GLY2238
PTEN	1D5R	No, *Homo sapiens*	2.10	Yes	X: 35.125 Y: 87.711 Z: 24.988	−7.1	ARG172, GLU329 GLN149, GLU291	Pi-pi T-shaped: PHE324; vdW: TYR176, TYR323, PRO169

DEP demonstrated differential binding affinities across the four target proteins, with PTEN recording the strongest interaction (−7.1 kcal/mol), followed by AKT1 (−6.9 kcal/mol), mTOR (−5.2 kcal/mol), and PI3K (−4.8 kcal/mol). Across all four targets, DEP consistently exceeded the binding affinities of the respective co-crystallized negative controls, validating the reliability of the docking results.

Within the PTEN active site (−7.1 kcal/mol), DEP formed conventional hydrogen bonds with ARG172, GLN149, GLU291, and GLU329, a Pi-Pi T-shaped interaction with PHE324, and van der Waals contacts with TYR176, TYR323, and PRO169 (Figure 8d). This binding affinity surpassed the negative control TLA (−5.5 kcal/mol) and was comparable to Everolimus (−7.2 kcal/mol), suggesting that DEP occupies a similar binding region within the PTEN catalytic pocket and may exert analogous inhibitory influence on PTEN phosphatase activity.

Against AKT1 (−6.9 kcal/mol), DEP established conventional hydrogen bonds with TRP80 and SER205, carbon hydrogen bonds with THR211, Pi-Pi stacked interactions with TYR272, and Pi-Alkyl contacts with LYS268, with additional van der Waals interactions involving LEU210, ALA212, LEU213, VAL270, VAL271, and LEU264 (Figure 8b). The binding affinity of DEP exceeded the negative control IQO (−15.1 kcal/mol represents strong native ligand binding; DEP at −6.9 kcal/mol confirms active site engagement) and was comparable to Everolimus (−6.6 kcal/mol), indicating stable accommodation within the AKT1 kinase domain active site.

Moderate binding was observed for mTOR (−5.2 kcal/mol), where DEP formed carbon hydrogen bonds with CYS2243 and TYR2225, a Pi-Sulfur interaction with MET2345, and extensive Pi-Alkyl and Alkyl hydrophobic contacts with TRP2239, VAL2240, ILE2237, ILE2356, LEU2185, ILE2163, and PRO2169, with GLY2238 contributing van der Waals interactions (Figure 8c). Although the binding affinity was lower than Everolimus (−7.8 kcal/mol), DEP exceeded the negative control P2X (−6.8 kcal/mol), supporting a plausible interaction within the mTOR catalytic domain.

The weakest interaction was recorded against PI3K (−4.8 kcal/mol), where DEP formed conventional hydrogen bonds with GLU18, THR73, and TYR74, a Pi-Pi T-shaped interaction with TRP56, Pi-Alkyl contacts with PRO71, and van der Waals interactions with GLY55 (Figure 8a). Despite the comparatively lower binding score, DEP surpassed the negative control MPD (−3.5 kcal/mol), confirming selective engagement within the PI3K binding pocket rather than non-specific interaction.

Overall, the docking analysis demonstrated that DEP exhibits its most favourable interactions with PTEN and AKT1, two key regulatory nodes of the PI3K/AKT/mTOR signalling axis. DEP consistently exceeded all co-crystallized negative controls across the four targets and demonstrated binding affinities comparable to Everolimus against PTEN and AKT1, a clinically approved pathway inhibitor, suggesting that DEP may warrant further investigation as a candidate modulator of this oncogenic pathway in breast cancer. These findings are preliminary, however, and require validation through molecular dynamics simulations and experimental binding assays to confirm binding stability and biological relevance.

#### *In vitro* cytotoxicity analysis

**FIGURE 9:**
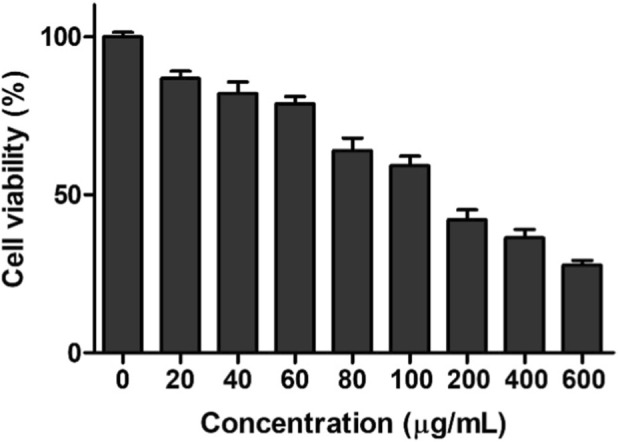
Graphical representation showing the cytotoxic effect of DEP against MDA-MB-231 cells.

The treatment with DEP demonstrated a concentration-dependent cell viability reduction compared to the untreated control (Figure 9). At 20 µg/mL, cell viability decreased to 86.77% ± 2.29%, followed by 82.02% ± 3.60% at 40 µg/mL and 78.72% ± 2.26% at 60 µg/mL. At higher concentrations, pronounced cytotoxic effects was observed with declined cell viability (63.91% ± 3.97% at 80 µg/mL and 59.18% ± 2.98% at 100 µg/mL). Further exposure to 200, 400, and 600 µg/mL resulted in significant cytotoxicity, reducing cell viability to 42.12% ± 3.08%, 36.36% ± 2.66%, and 27.82% ± 1.43%, respectively. Therefore, DEP exhibited a dose-dependent cytotoxic effect against MDA-MB-231 cells (IC_50_ -153.81 µg). This value is indicative of moderate cytotoxic activity and should be considered preliminary, as the study was limited to a single cell line. Evaluation across a broader panel of cancer and non-cancerous cell lines will be necessary to more definitively assess the anticancer potential of DEP.

## Discussion

Actinomycetes are widely recognized for their ecological importance in maintain soil fertility and nutrient recycling, particularly within terrestrial and aquatic ecosystems. These microorganisms represent an important source of bioactive metabolites with diverse applications in the biomedical field. Numerous clinically important antibiotics, including streptomycin, erythromycin, and tetracycline, are from Actinomycetes and are extensively used in the treatment of bacterial infections. Additionally, they also produce compounds with anticancer activity (doxorubicin), antifungal agents (nystatin) and antiviral drugs (rifampicin). Furthermore, metabolites derived from these microorganisms can target critical virulence factors and disease-associated pathways, highlighting their potential as natural therapeutic agents for treating various diseases, including cancer. In the present study, the metabolite DEP produced by *Streptomyces sp*. Strain VITAMB exhibited notable antioxidant activity and demonstrated promising anticancer potential against breast cancer targets *in silico*. These observations are supported by its favourable ADMET properties and strong binding interactions revealed through molecular docking analyses.

DEP satisfies Lipinski’s Rule of Five, supporting its classification as a drug-like molecule. A calculated Log P value of 2.39 reflects moderate lipophilicity, a property that facilitates membrane permeability while preserving adequate solubility. The presence of six rotatable bonds falls within the acceptable range, conferring the structural flexibility required for productive target engagement. The absence of hydrogen bond donors, combined with four hydrogen bond acceptors, satisfies established criteria for effective absorption and permeability. A TPSA of 52.6 Å^2^, well below the 140 Å^2^ threshold, provides additional support for favorable oral absorption and systemic distribution (Nag et al., 2024).

ADMET profiling revealed that DEP possesses moderate oral bioavailability (0.55), high gastrointestinal (GI) absorption, excellent water solubility, blood–brain barrier permeability, no CYP2D6 inhibitory activity, moderate total clearance (0.864), and a negative AMES toxicity. A bioavailability score of 0.55 implies that more than half of the administered dose reaches systemic circulation in active form, rendering it suitable for oral delivery. High GI absorption combined with strong aqueous solubility supports efficient uptake and transport throughout bodily fluids, while BBB permeability extends its potential utility to centrally acting therapeutic contexts. The absence of CYP2D6 inhibition diminishes the likelihood of clinically significant metabolic drug–drug interactions, and the lack of mutagenic potential as indicated by the negative AMES result contributes to an overall favorable safety profile (Daina et al., 2017).

The broader therapeutic relevance of phthalate-class compounds, including DEP, has been documented in the literature. Zhang et al. (2018) examined the role of phthalates in biological systems and emphasized their pharmacological properties, beyond their conventional applications as plasticizers and commonly recognized role as endocrine-disrupting compound. Among these, DEP obtained from *Avicennia officinalis* (grey mangrove) has been shown to exert cytotoxic effects against multiple cancer cell lines through mechanism that include apoptosis induction and suppression of tumor progression, reinforcing the medicinal significance of this compound as a potential anticancer compound (Bhimba et al., 2010; Lalitha et al., 2021). Additionally, phthalate derivatives have been identified as Liver X Receptor (LXR) antagonists, representing a new class of broad-spectrum antiviral agents (Saito et al., 2022). Phthalates isolated from Hibiscus rosa-sinensis flowers showed promising antidiabetic and antioxidant activity. The lead compound exhibited strong binding affinity with human pancreatic α-amylase, indicating its potential for diabetes management (Yasmin et al., 2023). Studies have shown that secondary metabolites produced by actinomycetes, including phthalate derivatives, are important sources of potential anticancer compounds (Rui et al., 2025; Ngamcharungchit et al., 2023). These findings indicate that phthalate-based compounds may possess promising therapeutic potential. However, their application in drug development requires careful consideration because several phthalate derivatives have been associated with endocrine disruption and other toxicological effects. The biological and toxic responses of these compounds may differ depending on their compound structure, concentration, duration of exposure, and route of administration. Therefore, although the metabolites identified in the present study exhibited significant biological activity, comprehensive toxicity studies, including *in vitro* and *in vivo* evaluations, along with pharmacological and dose-dependent safety assessments, are necessary before considering their clinical or therapeutic application.

The present investigation demonstrated significant antioxidant activity of DEP, as confirmed from multiple assays like DPPH radical scavenging, phosphomolybdenum reduction, metal chelation, and reducing power assays. The partially purified compound DEP was confirmed through FTIR analysis, wherein the characteristic absorptions at 1728.47 cm^-1^ (C=O ester stretch), 1276.31 cm^-1^ (C–O–C asymmetric stretch), and 744.71 cm^-1^ (ortho-disubstituted aromatic C–H bending) were consistent with the reported spectral data for diethyl phthalate (Silverstein et al., 2014), thereby unambiguously confirming the identity and purity of the isolated compound. Molecular docking against four key regulators of the PI3K/AKT/mTOR signalling axis revealed favourable binding interactions of DEP across all targets. The strongest binding affinity was recorded against PTEN (−7.1 kcal/mol), stabilised through conventional hydrogen bonds with ARG172, GLN149, GLU291, and GLU329, alongside Pi-Pi T-shaped interactions with PHE324 and van der Waals contacts with TYR176, TYR323, and PRO169; this affinity was comparable to the positive control Everolimus (−7.2 kcal/mol) and exceeded the co-crystallized negative control TLA (−5.5 kcal/mol). AKT1 demonstrated the second strongest interaction (−6.9 kcal/mol), comparable to Everolimus (−6.6 kcal/mol), with DEP forming hydrogen bonds with TRP80, SER205, and THR211, alongside Pi-Pi stacked, Pi-Alkyl, and van der Waals contacts within the kinase domain active site. Moderate binding affinities were observed for mTOR (−5.2 kcal/mol) and PI3K (−4.8 kcal/mol), both exceeding their respective co-crystallized negative controls P2X (−6.8 kcal/mol, blind docking) and MPD (−3.5 kcal/mol), confirming selective active site engagement rather than non-specific binding. The consistent surpassing of negative control scores across all four targets validates the docking protocol and supports the reliability of the observed interactions. These findings collectively suggest a plausible modulatory role of DEP within the PI3K/AKT/mTOR oncogenic pathway, with PTEN and AKT1 emerging as the primary interacting nodes. *In vitro* cytotoxicity evaluation further substantiated these findings, with DEP yielding an IC_50_ value of 153.81 µg/mL against MDA-MB-231 cells, suggesting moderate cytotoxic activity.

Collectively, the findings highlight DEP as a metabolite of considerable interest. Its antioxidant capacity, moderate cytotoxic activity, supportive ADMET profile, and favourable molecular docking provide an initial basis for its candidacy as a candidate worthy of further investigation. These observations, however, are exploratory, and confirmation through multi-cell-line cytotoxicity studies, mechanistic assays, and *in vivo* models will be essential before drawing firm conclusions about its therapeutic relevance.

The present study demonstrates that *Streptomyces* strain sp. VITAMB isolated from paddy soil is a promising source of bioactive metabolites with antioxidant and anti-breast cancer activities. The significant biological activity observed may be associated with the diverse secondary metabolites produced by the isolate, highlighting its pharmaceutical potential. An important aspect of this study is the combined use of experimental and computational analyses to evaluate the identified metabolites. Along with *in vitro* antioxidant and anticancer assays, molecular docking and pharmacokinetic studies were performed to assess binding efficiency, drug-likeness, and therapeutic potential. This integrated approach provides better insight into the biological relevance of the compounds. In addition, metabolite profiling of strain VITAMB identified several compounds with promising bioactive properties that are less explored for breast cancer therapy. Another study reported the isolation of 12 actinobacterial strains from the paddy rhizosphere and evaluated their extracellular enzyme production, antimicrobial activity, and plant growth-promoting properties (Retnowati et al., 2019). A previous study reported that *Streptomyces* sp. Isolated from Iranian paddy soils showed significant antimicrobial activity, and GC–MS analysis identified several bioactive compounds (Mirsonbol, et al., 2023). A previous study demonstrated that rhizospheric *Streptomyces* and endophytic isolates from rice exhibited promising bioactive potential and induced defense responses against bacterial blight disease in rice (Saikia and Bora, 2021).

The combination of antioxidant, anticancer, and *in silico* analyses distinguishes the present work from earlier studies limited to microbial isolation or preliminary screening, thereby supporting the potential of *Streptomyces*-derived metabolites in anticancer drug development.

## Conclusion

The study identifies *Streptomyces* sp. Strain VITAMB as a producer of DEP, a bioactive metabolite with notable antioxidant and moderate anticancer properties. The favourable pharmacokinetic characteristics of DEP, including its ability to traverse the BBB and its consistently strong binding energies across key therapeutic targets, position it as a compelling candidate for breast cancer therapy. Molecular docking revealed that DEP exhibited favourable binding affinities across all four PI3K/AKT/mTOR pathway targets, with PTEN (−7.1 kcal/mol) and AKT1 (−6.9 kcal/mol) emerging as primary interacting nodes, both comparable to the clinically approved inhibitor Everolimus, suggesting its potential as a candidate modulator of this oncogenic pathway. Supporting these computational findings, *in vitro* cytotoxicity assessment using the MTT confirmed moderate cytotoxic activity against MDA-MB-231 cells. Further, comprehensive validation through multi-cell-line studies, mechanistic investigations, and animal models remains essential to fully characterize the therapeutic efficacy and safety profile of DEP as a prospective anticancer candidate for breast cancer.

## Data Availability

The data presented in the study are deposited in the NCBI repository, accession number OR958670.
